# Effect of platelet inhibition with perioperative aspirin on survival in patients undergoing curative resection for pancreatic cancer: a propensity score matched analysis

**DOI:** 10.1186/s12893-021-01083-9

**Published:** 2021-02-22

**Authors:** E. Pretzsch, J. G. D’Haese, B. Renz, M. Ilmer, T. Schiergens, R. C. Miksch, M. Albertsmeier, M. Guba, M. K. Angele, J. Werner, H. Nieß

**Affiliations:** Department of General, Visceral, and Transplant Surgery, Ludwig-Maximilians-University Munich, Munich, Germany

**Keywords:** Actylsalicylic acid, Adjuvant aspirin, Micrometastasis, Circulating tumor cells, Pancreatic surgery

## Abstract

**Background:**

The importance of platelets in the pathogenesis of metastasis formation is increasingly recognized. Although evidence from epidemiologic studies suggests positive effects of aspirin on metastasis formation, there is little clinical data on the perioperative use of this drug in pancreatic cancer patients.

**Methods:**

From all patients who received curative intent surgery for pancreatic cancer between 2014 and 2016 at our institution, we identified 18 patients that took aspirin at time of admission and continued to throughout the inpatient period. Using propensity score matching, we selected a control group of 64 patients without aspirin intake from our database and assessed the effect of aspirin medication on overall, disease-free, and hematogenous metastasis-free survival intervals as endpoints.

**Results:**

Aspirin intake proved to be independently associated with improved mean overall survival (OS) (46.5 vs. 24.6 months, *p = 0.006), median disease-free survival (DFS) (26 vs. 10.5 months, *p = 0.001) and mean hematogenous metastasis-free survival (HMFS) (41.9 vs. 16.3 months, *p = 0.005). Three-year survival rates were 61.1% in patients with aspirin intake vs. 26.3% in patients without aspirin intake. Multivariate cox regression showed significant independent association of aspirin with all three survival endpoints with hazard ratios of 0.36 (95% CI 0.15–0.86) for OS (*p = 0.021), 0.32 (95% CI 0.16–0.63) for DFS (**p = 0.001), and 0.36 (95% CI 0.16–0.77) for HMFS (*p = 0.009).

**Conclusions:**

Patients in our retrospective, propensity-score matched study showed significantly better overall survival when taking aspirin while undergoing curative surgery for pancreatic cancer. This was mainly due to a prolonged metastasis-free interval following surgery.

## Background

In early stages of pancreatic cancer, surgical resection is the most important pillar of therapy. However, a significant proportion of patients will subsequently develop and succumb to distant metastases due to clinically undetectable micrometastasis at the time of surgery.

The “invasion-metastasis cascade” that a localized primary tumor undergoes in order to form metastases, is highly dependent on interactions with healthy cells and components from the surrounding tumor microenvironment [1]. It is highly inefficient concerning the very small proportion of circulating tumor cells (CTCs) that will successfully form overt metastases in distant organs. The vast majority of CTCs dies from sheer stress or elimination by immune cells [2, 3]. Platelets within the circulation collaborate closely with CTCs, protect them from natural killer cells, sustain epithelial–mesenchymal transition (EMT) signals and promote survival through paracrine signals [4]. Thrombocytosis is frequently associated with poor survival and inhibition of platelets can diminish metastasis formation [5, 6]. Thus, the importance of platelets in facilitating metastasis is undisputed [7].

Aspirin, which is a potent inhibitor of platelet functionality, has been shown to positively affect cancer incidence and cancer-related mortality as reported in epidemiological studies and meta-analysis of randomized controlled trials for cardiovascular prevention [8–11]. However, only recently randomized controlled trials assessing the effect of aspirin on cancer survival were initiated [12, 13], none of which assess the effect of aspirin in the setting of pancreatic cancer surgery.

Despite the overall positive effect that resection of the primary tumor has on the patient [14], surgical trauma and physiological changes in the perioperative phase may negatively influence oncological outcome by promoting metastasis formation [15]. In particular, perioperative immunosuppression can lead to a metastasis-permissive state by hampering the main eliminator of circulating cancer cells, the cell-mediated immunity [16]. The perioperative phase represents a short but highly vulnerable phase of the overall disease course in cancer patients. Interference with its pro-metastatic aspects could thus positively affect long-term outcome. We sought to assess this effect by examining patients who continuously took aspirin before, during and after surgery and thus inhibited the main partners in crime of CTCs: platelets.

## Methods

### Study population and propensity score matching

The institutional review board of the University of Munich (study #20-101) approved the study protocol. Patients undergoing pancreatic surgery at our institution between 01/2014 and 11/2016 were registered in a prospectively maintained database. The study period was chosen because in 2014 a change of the department head and standards in pancreatic surgery took place at our institution. We retrospectively identified patients for our study population from this database using a pre-defined study protocol that was designed to address the research question. Inclusion criteria were: (1) confirmed diagnosis of pancreatic ductal adenocarcinoma (PDAC) by pathology, (2) exclusion of distant metastasis at time of surgery, (3) successful resection of primary tumor with curative intent, (4) postoperative survival and/or follow-up of at least 12 weeks, (5) disease-free survival of at least 6 weeks. Patients with diagnosis other than PDAC were excluded as well as patients that suffered from locally advanced disease that underwent neoadjuvant therapy. All PDAC in the database were staged and documented by a pathologist according to the 7th edition AJCC TNM criteria from 2010 [17]. Since we wanted to focus our analysis on the metastases that might be favored by the perioperative period, we excluded patients who developed metastases within the first 6 weeks after resection, as these must have presumably already existed at the time of surgery. We identified 18 patients who were already taking 100 mg aspirin daily at the time of in-patient admission for surgery as part of their regular medication for different indications and who were not paused for this medication at any time during their in-patient stay.

### Variables

The following variables were extracted from the database as possible confounders: age, sex, American Society of Anesthesiologist (ASA) score, body mass index (BMI), preoperative serum level of carboanhydrate antigen 19-9 (CA19-9), preoperative platelet count, type of resection, intraoperative blood transfusions received, necessity for vascular resection, TNM stage, maximum tumor diameter, examined lymph nodes, lymph node ratio (number of positive nodes divided by resected nodes), residual tumor status (R0 wide, R0 narrow, R1; according to [18], tumor grading, perineural, vascular, and lymphovascular invasion, and administration of adjuvant therapy.

Follow-up data was acquired by querying the Munich tumor register, by detailed analysis of the electronic patient files for most recent hospital visits and by contacting patients by telephone.

Overall survival (OS) and disease-free survival (DFS) were determined as time interval between day of surgery and day of death (OS)/last follow-up or diagnosis of recurrence (DFS)/last follow-up, respectively. Patients alive or recurrence-free, respectively, at last follow-up visit were censored in the analysis. Patients, in which death from disease was documented but no clear prior date of recurrence diagnosis could be determined, date of recurrence was set to the date of death for DFS. Since our hypothesis foresees an effect of perioperative aspirin specifically on hematogenous metastasis, we analyzed the time interval between surgery and first diagnosis of hematogenous metastasis as “hematogenous metastasis free survival (HMFS)”. Patients that died from other or unknown cause were censored at time of death from cancer specific survival analysis (DFS and HMFS). Patients that died with diagnosis of local recurrence or peritoneal carcinomatosis but without hematogenous metastasis were censored only in HMFS but not DFS.

### Propensity score matching

To reduce bias due to confounding variables, propensity score matching (PSM) was performed. PS was estimated as the predicted probability of a patient being on aspirin from a logistic regression model, considering variables that were available prior to surgery and potentially of prognostic value. PSM included T stage, age, sex, ASA score, and tumor diameter. We then formed matched pairs between the 18 patients from the aspirin group and the control group without aspirin using a one-to-four greedy nearest neighbor match without replacement with a caliper width of 0.5 (maximum allowable difference in propensity scores) (SAS software, Cary, NC, USA). The logit of propensity score was used as distance metric and the effective caliper of the matching process was 0.176. Four control patients for each patient of the therapy group were not available under these matching conditions. Thus, the matching process resulted in 18 patients in the therapy group matched to 64 in the control group (effectively 1:3.6 ratio). Good variable balance achieved by the matching process was assured with a treated-to-control variance ratio of the logit of the propensity score of 1.44, which lies between 0.5 and 2 as proposed by Rubin [19]. Only patients matched with PS were included in the time-to-event analyses.

### Statistical methods

Propensity score matching was performed using SAS, all further analyses were conducted using SPSS© Statistics (version 20, IBM©). Continuous variables were expressed as median with interquartile range (IQR). To detect differences in continuous variables, medians were compared using Mann–Whitney-U test. Potential differences of confounders measured as categorical variables were assessed using cross tabulation and Fisher’s exact-testing. All univariate survival analyses were conducted using Kaplan–Meier estimates and groups compared with log-rank tests. Prior to survival analysis, continuously expressed confounders were categorized by dichotomizing at the median. Two-sided p-values below 0.05 were considered statistically significant. Median, 3- and 5-year survival rates were reported when available. In groups where the number of events was too low to calculate a median survival, mean survival was reported.

Confounding variables that showed influence on the survival estimates (OS, DFS, HMFS) with a p-value of ≤ 0.10 were entered into a multivariate cox regression analysis from which hazard ratios, 95% confidence intervals and two-sided p-values were reported. Parameters with missing data (e.g. adjuvant therapy) were included in the multivariate analysis by adding the category “unknown” to the respective variable to prevent listwise exclusion of cases from multivariate analysis.

## Results

### Baseline characteristics and tumor stage

Median age of patients was 71 years (IQR: 62–75) and the female to male ratio was 35%:65%. Sixty-seven percent of patients received a pancreaticoduodenectomy, followed by distal pancreatectomy in 22% of patients and total pancreatectomy in 11% of patients. As defined per study protocol and confirmed by histology, all patients suffered from ductal adenocarcinoma of the pancreas. The predominant histopathological features were T3 tumor stage (93% of patients), poor differentiation (G3: 71% of patients) and presence of perineural invasion (Pn1: 87% of patients). Seventy-eight percent of patients showed clear resection margins after surgery (R0). Further patient characteristics are presented in Table 1.

**Table 1:** Baseline characteristics for the study population

Parameter	All (n = 82)	No ASS (n = 64)	ASS (n = 18)	*p*
Age^a^	71.1 [62.1–75.1]	71.6 [62.1–76]	70.6 [64–72.1]	0.779
Sex				1.0
Female	29 (35.4%)	23 (35.9%)	6 (33.3%)	
Male	53 (64.6%)	41 (64.1%)	12 (66.7%)	
ASA score				0.09
ASA II	16 (19.5%)	11 (17.2%)	5 (27.8%)	
ASA III	65 (79.3%)	53 (82.8%)	12 (66.7%)	
ASA IV	1 (1.2%)	0 (0%)	1 (5.6%)	
BMI	24.2 [22.1–26]	24.3 [22.5–26.1]	23.2 [21.9–26]	0.398
Serum CA 19–9 (U/ml)^a^	88.4 [27–368]	96.7 [27.3–381.]	49.5 [13.5–339]	0.292
Platelet count^a^ (G/l)	232 [178–279]	235 [177–310]	225 [181–272]	0.760
Type of surgery				0.474
PD	55 (67.1%)	45 (70.3%)	10 (55.6%)	
DP	18 (22%)	13 (20.3%)	5 (27.8%)	
TP	9 (11%)	6 (9.4%)	3 (16.7%)	
RBC during surg. (units)^a^				0.404
Yes	9 (11%)	6 (9.4%)	3 (16.7%)	
No	73 (89%)	58 (90.6%)	15 (83.3%)	
Additional venous resection				0.778
Yes	25 (30.5%)	19 (29.7%)	6 (33.3%)	
No	57 (69.5%)	45 (70.3%)	12 (66.7%)	
Tumor diameter max (cm)^a^	3.3 [2.5–4.3]	3.2 [2.5–4.4]	3.3 [2.3–4.2]	0.771
T stage				
T1 + 2	6 (7.3%)	4 (6.2%)	2 (11.1%)	0.608
T3	76 (92.7%)	60 (93.8%)	16(88.9%)	
N stage				1.0
N0	25 (30.5%)	20 (31.2%)	5 (27.8%)	
N1	57 (69.5%)	44 (68.8%)	13 (72.2%)	
ELN^a^	26 [19–35]	25 [19–34]	33 [27–39]	*0.007
LNR^a^	0.07 [0.0–0.16]	0.09 [0.0–0.17]	0.04 [0.0–0.08]	0.754
Grading				
G1	1 (1.2%)	1 (1.6%)	0 (0%)	
G2	23 (28%)	17 (26.6%)	6 (33.3%)	
G3	58 (70.7%)	46 (71.9%)	12 (66.7%)	
Perineural invasion				0.246
Pn0	11 (13.4%)	7 (10.9%)	4 (22.2%)	
Pn1	71 (86.6%)	57 (89.1%)	14 (77.8%)	
Vascular invasion				1.0
V0	68 (82.9%)	53 (82.8%)	15 (83.3%)	
V1	14 (17.1%)	11 (17.2%)	3 (16.7%)	
Lymphovascular invasion				0.282
L0	49 (59.8%)	36 (56.2%)	13 (72.2%)	
L1	33 (40.2%)	28 (43.8%)	5 (27.8%)	
R status				0.457
R0 wide	29 (35.4%)	24 (37.5%)	5 (27.8%)	
R0 narrow	35 (42.7%)	25 (39.1%)	10 (55.6%)	
R1	18 (22%)	15 (23.4%)	3 (16.7%)	
Adjuvant therapy				0.282
Yes	48 (58.5%)	36 (56.2%)	12 (66.7%)	
No	8 (9.8%)	8 (12.5%)	0 (0%)	
Unknown	26 (31.7%)	20 (31.2%)	6 (33.3%)	

Differences in baseline characteristics were assessed by Fisher’s exact test for categorical and Mann–Whitney-U test for continuous variables

*ASA* American Society of Anesthesiologists Score, *BMI* body mass index, *PD* pancreaticoduodenectomy, *DP* distal pancreatectomy, total pancreatectomy, *RBC* packed red blood cells, *ELN* examined lymph nodes, *LNR* lymph node ratio

^a^Values expressed as median [interquartile range]

*p < 0.05

### Follow-up data

Median follow up for all patients of this study was 20.3 months (IQR: 12.2–31.2 months), with death occurring in 51 patients (62.2%), tumor recurrence of any kind occurring in 64 patients (78%) and development of hematogenous metastasis in 50 patients (61%) during the follow-up period. Median overall survival, disease-free survival, and hematogenous metastasis-free survival were 24.9 months (95% CI 19.0–30.7), 13.6 months (95% CI 10.9–16.3), and 18.3 months (95% CI 13.0–23.7). Three- and five-year survival rates for the whole study population were 34.7% and 23.5%. We were able to confirm routine administration of adjuvant chemotherapy in 59% of all patients with available data on the administration of chemotherapy. Reasons for the omission of chemotherapy were patient choice or poor performance status. In 32% of all patients, we were unable to obtain information on whether or not the recommended adjuvant chemotherapy was administered.

### Effect of aspirin on survival parameters

None of the baseline characteristics showed significant differences between groups except for the number of examined lymph nodes (Table 1). In patients with aspirin intake, a median of 33 lymph nodes (IQR: 27–39) was resected and examined compared to a median of 22 resected and examined lymph nodes (IQR: 19–34) in patients without aspirin intake (*p = 0.007). However, the number of examined lymph nodes did not show a significant influence on any of the survival parameters (see Table 2).

**Table 2:** Results from univariate survival analyses of aspirin intake and confounders on overall (OS), disease-free (DFS), and hematogenous metastasis-free (HMFS) survival

Parameter	Median (mean^a^) OS (95% CI) in months	3-YSR	5-YSR	Log-rank *p*	Median (mean^a^) DFS (95% CI) in months	Log-rank *p*	Median (mean^a^) HMFS (95% CI) in months	Log-rank *p*
Pt. on aspirin				*0.006		*0.001		*0.005
Yes	46.5^a^ (35.6–57.4)	61.1%	61.1%		26.0 (16.7–35.3)		41.9^a^ (30.3–53.4)	
No	24.6^a^ (20.2–28.9)	26.3%	n/a		10.5 (6.5–14.5)		16.3 (11.4–21.1)	
Age				0.405		0.348		0.957
≤ Median	26.5 (16.3–36.8)	40.9%	26.2%		14.5 (8.2–20.8)		17.9 (12.1–23.8)	
> Median	24.9 (14.8–34.9)	27.4%	n/a		13.5 (11.2–15.8)		21.5 (11.4–31.5)	
Sex				0.555		0.856		0.949
Female	24.2 (16.2–32.2)	29.2%	n/a		13.9 (11.7–16.1)		18.3 (7.3–29.4)	
Male	27.1 (17.3–37.0)	36.7%	21.4%		13.5 (8.5–18.5)		19.7 (14.4–24.9)	
BMI				0.127		0.601		0.956
≤ Median	35.0 (19.4–50.6)	47.9%	28.9%		13.5 (6.7–20.3)		19.6 (13.6–25.7)	
> Median	21.0 (17.3–24.6)	40.3%	24.3%		13.9 (10.5–17.3)		17.7 (7.3–28.1)	
ASA score				0.702		0.754		0.596
ASA II	26.5 (17.7–35.4)	26.7%	n/a		14.5 (10.9–18.1)		14.5 (10.9–18.1)	
ASA III/IV	22.3 (14.4–30.2)	36.2%	21.2%		13.3 (9.7–16.9)		20.9 (12.7–29.0)	
Platelet count				0.598		0.720		0.616
Normal	24.2 (17.6–30.8)	34.0%	n/a		13.6 (11.0–16.2)		17.9 (11.4–24.4)	
< 150 G/l	21.0 (9.8–32.2)	n/a	n/a		9.0 (7.8–10.2)		23.9^a^ (14.8–32.9)	
> 400 G/l	37.8 (0–81.7)	66,7%	n/a		19.7 (0.8–38.6)		19.7 (0.8–38.5)	
Serum CA19-9				0.092		0.128		0.196
≤ Median	29.1 (19.3–38.8)	41.8%	n/a		15.5 (12.3–18.7)		26.0 (15.0–37.0)	
> Median	18.9 (11.3–26.5)	26.5%	21.2%		12.0 (7.0–17.0)		18.3 (10.7–25.9)	
Type of resection				0.314		0.995		0.999
PD	21.0 (17.7–24.2)	29.4%	21.5%		12.8 (9.3–16.3)		18.3 (9.2–27.4)	
DP	37.1 (21.6–52.6)	52.1%	n/a		16.2 (14.7–17.7)		20.9 (14.4–27.3)	
TP	26.5 (22.4–30.7)	30.0%	n/a		14.5 (7.3–21.7)		16.8 (12.1–21.4)	
Tumor diameter				0.150		0.059		*0.032
≤ Median	27.0 (17.4–36.6)	41.7%	37.1%		13.9 (8.8–19.0)		27.0 (17.6–36.4)	
> Median	22.0 (13.4–30.6)	28.2%	n/a		12.8 (7.0–18.6)		16.2 (12.9–19.4)	
T-stage				0.352		0.249		0.402
T1/2	37.8 (13.6–62.0)	60.0%	30.0%		16.7 (12.5–20.9)		19.7 (12.8–26.5)	
T3	24.2 (17.4–31.0)	32.8%	n/a		13.3 (10.3–16.3)		17.9 (11.4–24.5)	
Nodal stage				0.491		0.909		0.601
N0	18.9 (13.9–23.9)	25.2%	25.2%		12.5 (9.1–15.9)		16.2 (8.1–24.2)	
N1	26.5 (21.2–31.8)	39.5%	n/a		13.6 (10.4–16.8)		18.3 (11.8–24.8)	
ELN				0.120		0.103		0.540
≤ Median	21.8 (15.7–27.9)	28.6%	n/a		12.8 (9.0–16.6)		19.7 (14.3–25.0)	
> Median	27.1 (15.6–38.6)	40.6%	34.8%		14.9 (9.3–20.5)		17.7 (2.8–32.6)	
Grading				0.840		0.713		0.240
G1/2	35.0 (9.3–60.8)	41.0%	n/a		16.2 (12.1–20.3)		27.2 (12.4–41.9)	
G3	24.2 (18.3–30.1)	32.9%	24.0%		12.5 (9.0–16.0)		17.7 (11.6–23.8)	
Lymphovasc. invas				*0.039		0.095		*0.049
L0	27.4 (18.8–36.0)	42.1%	31.2%		16.3 (13.8–18.8)		26.0 (18.0–34.0)	
L1	18.9 (10.1–27.7)	24.6%	n/a		9.0 (7.2–10.8)		13.9 (8.6–19.2)	
Vascular invasion				*0.039		**< 0.001		**< 0.001
V0	27.0 (18.2–35.8)	38.7%	25.7%		16.3 (12.4–20.2)		24.9 (17.3–32.4)	
V1	14.4 (12.1–16.6)	16.3%	n/a		7.9 (6.4–9.4)		9.0 (4.5–13.6)	
Perineural invasion				0.424		0.153		0.216
Pn0	35.0 (11.1–58.9)	40.4%	20.2%		27.8 (11.9–43.7)		32.7 (17.4–48.0)	
Pn1	24.2 (17.2–31.2)	33.6%	n/a		13.3 (10.3–16.3)		17.7 (12.2–23.2)	
R-status				0.366		0.756		0.225
R0 wide	26.5 (18.3–34.8)	35.5%	24.3%		14.9 (11.2–18.6)		21.5 (11.4–31.5)	
R0 narrow	29.1 (15.8–42.3)	39.6%	n/a		13.9 (9.6–18.2)		17.9 (9.6–26.3)	
R1	18.9 (6.0–31.8)	26.7%	n/a		12.0 (4.6–19.4)		12.5 (1.2–23.7)	
Add. venous resect				0.248		0.494		0.994
Yes	17.6 (6.0–29.1)	25.0%	n/a		12.5 (6.3–18.7)		18.3 (9.3–27.4)	
No	27.1 (19.0–35.2)	38.7%	22.7%		13.9 (11.6–16.2)		19.7 (12.7–26.6)	
Adjuvant therapy				0.010		0.634		0.672
Yes	27.1 (14.2–40.1)	43.1%	n/a		15.5 (12.2–18.8)		19.7 (12.3–27.0)	
No unknown	12.4 (8.6–16.2) 19.3 (12.4–26.2)	n/a 26.9%	n/a 13.4%		8.3 (7.5–9.1) 12.8 (8.3–17.3)		21.5 (4.9–38.1)	

*BMI* body mass index, *ASA* American Society of Anesthesiologists Score, *PD* pancreaticoduodenectomy, *DP* distal pancreatectomy, total pancreatectomy; *ELN* examined lymph nodes

^a^Median could not be calculated due to too many censored cases, mean survival is presented; patients with missing data were excluded in univariate analyses for the respective parameter

*p < 0.05, **p ≤ 0.001

**Fig. 1:**
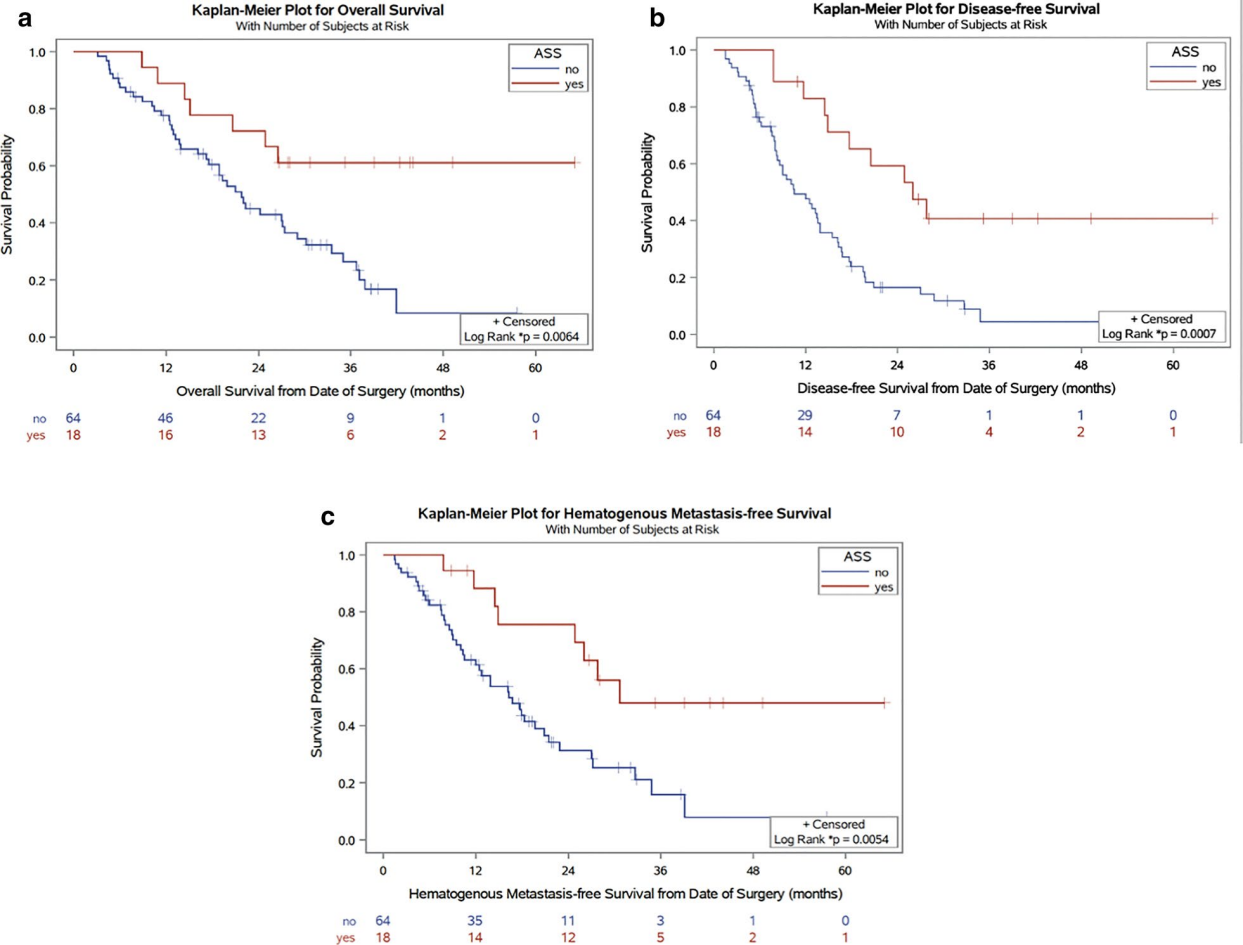
Overall, disease-free, and hematogenous metastasis-free survival in patients following curative pancreatic surgery with (red line) and without aspirin (blue line). Depicted is **a** overall survival, **b** disease-free survival, and **c** hematogenous metastasis free survival (HFMS) in months following pancreatic surgery. p-values were calculated by log-rank test

Univariate analysis by Kaplan–Meier estimates and log-rank test showed that aspirin intake was associated with longer mean OS compared to no aspirin intake (46.5 vs. 24.6 months, *p = 0.006), longer DFS (26.0 vs. 10.5 months, *p = 0.001) and longer mean HMFS (41.9 vs. 16.3 months, *p = 0.005) (Fig. 1, Table 2).

Other parameters that were significantly associated with longer OS were absent lymphovascular invasion (27.4 vs. 18.9 months, *p = 0.039), absent vascular invasion (27.0 vs. 14.4 months, *p = 0.039) and administration of adjuvant chemotherapy (27.1 vs. 12.4 months, *p = 0.010). Another parameter that showed significant difference in median disease-free survival was absent vascular invasion (16.3 vs. 7.9 months, **p < 0.001). Parameters that were significantly associated with longer hematogenous metastasis free survival were tumor diameter below median (27.0 vs. 16.2 months, *p = 0.032), absent lymphovascular invasion (26.0 vs. 13.9 months, *p = 0.049) and absent vascular invasion (24.9 vs. 9.0 months, **p < 0.001) (Table 2).

All parameters with a p-value of 0.10 or below in univariate analysis were subsequently tested in a multivariate analysis using Cox regression (Table 3). Among all parameters, only aspirin intake was independently associated with better OS (HR 0.36, *p = 0.023), DFS (HR 0.32, **p = 0.001) and HMFS (HR 0.36, *p = 0.010).

**Table 3:** Results from multivariate cox regression of survival parameters

Parameter influencing overall survival	HR	95% CI	*p*
Pt. on aspirin (yes vs. no)	0.357	0.149–0.856	*0.021
Not included			
Vascular invasion (yes vs. no)			0.228
Serum CA19-9 above median			0.490
Lymphovascular invasion			0.440
Adjuvant chemotherapy			0.061
**Parameter influencing disease-free survival**			
Pt. on aspirin (yes vs. no)	0.316	0.158–0.634	**0.001
Vascular invasion (yes vs. no)	3.008	1.541–5.871	**0.001
Not included			
Tumor diameter (> median vs. ≤ median)			0.081
Lymphovascular invasion			0.484
**Parameter influencing hematogenous-met-free survival**			
Pt. on aspirin (yes vs. no)	0.354	0.162–0.772	*0.009
Vascular invasion (yes vs. no)	4.330	2.006–9.344	**< 0.001
Not included			
Tumor diameter (> median vs. ≤ median)			0.065
Lymphovascular invasion			0.258

Vascular invasion was independently associated with decreased overall survival (HR 3.01, **p = 0.001) and reduced hematogenous metastasis free survival (HR 4.80, **p < 0.001) (Table 3).

## Discussion

Patients suffering from pancreatic cancer have a high risk for recurrence and death despite curative resection with the majority of patients succumbing to diffuse metastatic disease. In fact, 78% of the patients of our curatively resected cohort developed a relapse in the follow-up period of our study and 60.6% died. Our survival results closely resemble those reported in the gemcitabine arm of ESPAC-4, which was the most recent multicentric randomized controlled trial with adjuvant gemcitabine use at the time our patients were treated (OS: 25 vs. 25.5 months, DFS: 13.6 vs. 13.1 months) [20]. Most prognostically relevant baseline characteristics were also comparable to those enrolled in ESPAC-4 [e.g. similar R0 rate (35.4% vs. 40%), maximum tumor diameter (33 mm vs. 30 mm), T3 stage (92.7% vs. 89%)]. Several of the confounders that influenced survival in our study cohort have previously been described to do so in the literature [21, 22].

Aspects related to surgery, such as immunosuppression, release of tumor-supporting factors, and increase of circulating tumor cells (CTCs) by manipulation presumably favor the probability of distant metastases [15]. Platelets have been attributed important roles in increasing the metastatic potential of CTCs by associating with them via tissue factor on the surface of cancer cells [23]. Not only does this screen cancer cells from natural killer cell-mediated killing, it also enables them to benefit from the bioactive molecules abundantly contained in and released by platelets (e.g. TGF-ß and PDGF) [23–25]. These molecules cause normal somatic cells, e.g. immune and endothelial cells, to act in favor for the metastatic process [4]. They also support the continuation of EMT pathway activation in CTCs, an important prerequisite for metastatic seeding and otherwise abrogated after losing contact with stromal signals in the primary tumor [26]. Thus, we speculated whether aspirin could counteract negative aspects of the surgical procedure and have a positive impact on patients in this vulnerable phase.

For many decades, aspirin was paused before surgery out of concern for excessive bleeding. This practice has changed only in recent years in our institution out of worry for increased cardiovascular events during surgery, especially owing to the temporarily increased activity of thromboxane A2 after discontinuation of aspirin ("aspirin-withdrawal syndrome") [27]. While the decision to continue aspirin was not based on oncological reasons, it now allows us to assess the clinical outcomes in this patient cohort. In order to be able to detect an effect of perioperative aspirin at all, we considered it important to include only patients in the therapy group who took aspirin permanently and without pausing.

Weighing up potential risks of aspirin, it is known to cause an increase in absolute risk for major bleeding in the general population from about 0.16% to 0.81% over a 15-year period [28]. Most surgical studies, however, show no relevant increase in bleeding risk for patients on aspirin during the perioperative phase and demonstrate a generally high level of safety for the drug [29]. This is consistent with our observations, given that the rate of perioperative blood transfusions was evenly low in both groups of our study.

To our knowledge, there is no published data from randomized phase 3 studies that examine the efficacy of aspirin as cancer therapeutic for pancreatic cancer as an endpoint. However, there are manifold data from experimental, epidemiological, and observational studies, which support the hypothesis of an anti-cancer effect of aspirin [30]. In pre-clinical studies, aspirin has been shown to counteracts cancer stem cell features, desmoplasia and gemcitabine resistance, which was the adjuvant chemotherapy used in in our patient cohort [31, 32]. Furthermore, aspirin reduced Foxp3+ regulatory T cells and prolonged survival in a mouse model and showed effects on Cox-2 expressed in cancer cells, which is involved in carcinogenic pathways (i.e. RAS and NF-κB) [33].

Metaanalyses of large cohort studies have linked regular aspirin intake with a reduced incidence of various cancers, including colorectal, gastric and prostate cancer [30]; while research into pancreatic cancer has yielded inconclusive results [34, 35]. Additionally, randomised controlled trials in cardiovascular disease suggest that aspirin may lead to a marked reduction in the mortality associated with several cancer entities. This may be due to a (50%) overall reduction in the occurence of metastases. Further, in patients who primarily develop non-metastatic adenocarcinoma, aspirin reducedthe risk of susequent metastasis formation by approximately 70% [10]. Patients from our study that continuously took aspirin showed significantly longer disease-free and hematogenous metastasis-free intervals (Table 3), which resulted in improved overall survival.

Due to the non-randomized nature and the small sample size of our study, it certainly does not prove that the observed survival benefit is in fact caused by aspirin use. Instead, if aspirin indeed proves to be effective against pancreatic cancer, long-term administration of aspirin could just as well explain the survival benefit observed in our study population by providing continuous protection against the formation of metastases from CTCs prior to surgery or residual tumor cells after surgery. It is however plausible from the suspected mechanism of action and cancer-favoring risks of the surgical procedure, that aspirin intake might be of particular importance in the perioperative setting. Only a prospective randomized controlled trial where patients in the therapy arm are started on aspirin as soon as the diagnosis of pancreatic cancer is suspected, and the medication is continued along with adjuvant chemotherapy until the end of the study could provide answers hereto. In some tumor entities (e.g. colorectal carcinoma) aspirin may exert beneficial effects only after several years of administration, which may reflect the halting effect of aspirin on carcinogenesis, wherease recent studies could demonstrate that aspirin might also provide shortterm benefits by inhibition of metastasis formation [10, 36, 37].

However, despite these broad indications for the efficacy of aspirin in pancreatic cancer or even cancer in general and despite the drug being used safely for many decades in millions of patients, clinical data on the efficacy against cancer is surprisingly sparse.

## Conclusion

Our propensity score matched study demonstrates that patients undergoing curative resection of pancreatic adenocarcinoma with continuous perioperative aspirin intake show significantly better survival endpoints than patients without aspirin intake. Taken together with pre-existing evidence, there is sufficient indication of efficacy to justify testing this hypothesis in a prospective randomized study.

## Data Availability

The data that support the findings of this study are available on request from the corresponding author. The data are not publicly available due to privacy or ethical restrictions.

## Abbreviations

ASA: American Society of Anesthesiologist
BMI: Body mass index
CA19-9: Carboanhydrate antigen 19-9
CTCs: Circulating tumor cells
DFS: Disease-free survival
EMT: Epithelilal–mesenchymal transition
HMFS: Hematogenous metastasis-free survival
OS: Overall survival
PDAC: Pancreatic ductal adenocarcinoma
PSM: Propensity score matching
